# *In vitro* and *in vivo* biocompatibility and inflammation response of methacrylated and maleated hyaluronic acid for wound healing

**DOI:** 10.1039/d0ra06025a

**Published:** 2020-08-28

**Authors:** Lijun Zhang, Ugo D'Amora, Alfredo Ronca, Yuanyuan Li, Xiaoying Mo, Fei Zhou, Mingzhou Yuan, Luigi Ambrosio, Jun Wu, Maria Grazia Raucci

**Affiliations:** Department of Burn and Plastic Surgery, Shenzhen Second People's Hospital, The First Affiliated Hospital of Shenzhen University Shenzhen P. R. China junwupro@126.com; Institute of Polymers, Composites and Biomaterials, National Research Council Naples Italy alfredo.ronca@cnr.it; Department of Burns, The First Affiliated Hospital, Sun Yat-sen University Guangzhou P. R. China

## Abstract

Over the past few years, different *in vitro* and *in vivo* studies have been highlighting the great potentiality of hyaluronic acid (HA) as a biomaterial in wound healing treatment thanks to its good capability to induce mesenchymal and epithelial cell growth and differentiation, angiogenesis, and collagen deposition. However, the need to improve its mechanical properties as well as its residence time has led scientists to study new functionalization strategies. In this work, chemically modified HA-based hydrogels were obtained by methacrylic and maleic functionalization. Methacrylated (MEHA) and maleated HA (MAHA) hydrogels have shown important physico-chemical properties. The present study provides a deeper insight into the biocompatibility of both synthesized materials and their effects on tissue inflammation using *in vitro* and *in vivo* models. To this aim, different cell lines involved in wound healing, human dermal fibroblasts, human adipose-derived stem cells and human umbilical vein endothelial cells, were seeded on MEHA and MAHA hydrogels. Furthermore, an inflammation study was carried out on a murine macrophage cell line to assess the effects of both hydrogels on inflammatory and anti-inflammatory interleukin production. The results showed that both MAHA and MEHA supported cell proliferation with anti-inflammation ability as highlighted by the increased levels of IL-10 (57.92 ± 9.87 pg mL^−1^ and 68.08 ± 13.94 pg mL^−1^, for MEHA and MAHA, respectively). To investigate the inflammatory response at tissue/implant interfaces, an *in vivo* study was also performed by subcutaneous implantation of the materials in BALB/c mice for up to 28 days. In these analyses, no significant chronic inflammation reaction was demonstrated in either MEHA or MAHA in the long-term implantation.

## Abstract 2

From synthesis to the *in vitro* and *in vivo* biological evaluation of two types of hyaluronan derivatives.

## Graphical abstract

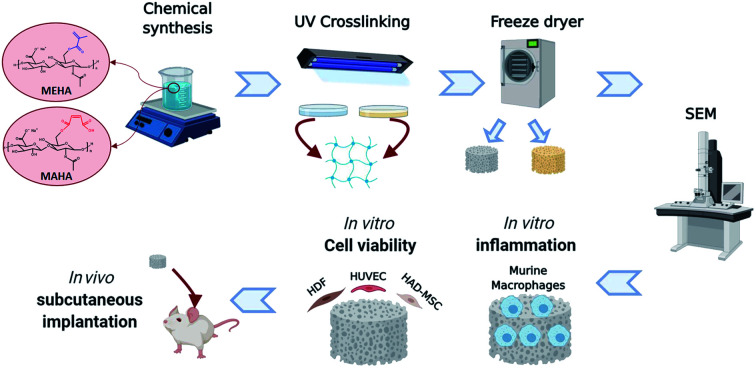

## 1. Introduction

Hydrogels are hydrophilic polymer networks capable of absorbing a high quantity of water, up to thousands of times their dry weight.^1–3^ Due to their hydrophilic nature, they allow diffusion of small molecules, nutrients and oxygen, providing an ideal 3D microenvironment for cell proliferation and differentiation.^4,5^ Since hydrogels have structural similarities with the extracellular matrix (ECM), they have been extensively used in various biomedical applications as drug delivery agents,^6^ bio-adhesives,^7^ and as scaffolds for regenerative medicine.^8–10^ Among natural hydrogels, hyaluronic acid (HA) also called hyaluronan, plays a central role in maintaining cell and tissue integrity, promoting cell proliferation, intracellular signaling and wound repair.^11^ HA is a linear polysaccharide, consisting of alternating β-1,4-linked units of β-1,3-linked glucuronic acid and *N*-acetyl-d-glucosamine suitable for formulating hydrogels.^12^ It is the main constituent of the ECM in human connective tissue and allows a structural assembling of aggrecan components.^13–17^ Thanks to its biological properties such as biocompatibility and being non-allergenic, HA represents a suitable material for application to the skin or dermis layer.^18–21^ Furthermore, benefiting from its physico-chemical performances such as unique water retention capacity, hydrophilicity, rheological, and viscoelastic behavior, HA affects cellular response in terms of cell attachment, growth, migration and differentiation.^13,22^ Indeed, several *in vitro* and *in vivo* studies have demonstrated the good potential of HA in wound healing treatment by inducing mesenchymal and epithelial cell migration and differentiation, improved angiogenesis, and collagen deposition.^23^ Furthermore, it has been described that metabolic degradation by-products of HA are able to stimulate endothelial cell proliferation and migration, tailoring the inflammatory processes and angiogenesis at different stages of wound healing.^18,22,24^ In particular, HA can be recognized by CD44, a cell surface receptor that regulates cell adhesion, growth and differentiation, and all the physiological processes such as immune response, wound healing and vascularization.^25^ Taking into consideration all these properties, HA-based scaffolds/patches have also been developed and analyzed in both fundamental science and clinical applications,^13,26^ including tissue engineering,^15^ cosmetic,^27^ cell and drug delivery^28^ and bio-printing.^29,30^ However, the main issues limiting its applicability as biomaterial are the high degradation rate, uncontrolled swelling in physiological condition and low mechanical stability.^31^ This affects stem cell differentiation and consequently tissue regeneration capacity.^32,33^ To overcome these issues, chemical crosslinking of HA, by chemical modifications, has been widely studied.^8,34–37^ In particular, such chemical modifications allow to increase degradation time and improve stability in physiological environment.^3^ A wide spectrum of chemically different HA-based products has been reported in literature with different physico-chemical properties.^34,38–40^ Li *et al.* developed HA-based patches using HA grafted pullulan (HA-*g*-Pu) showing that this material has higher swelling ratio (40%) in comparison to HA (34%) and pullulan (30%), and an extended degradation profile (up to 12–14 days).^41^ Successively, Duan *et al.* synthesized a series of curcumin grafted HA-modified pullulan, which showed good biocompatibility, antimicrobial, antioxidant and wound healing properties.^42^ However, interesting results were also obtained by combining HA with other polymers such as silk fibroin,^43^ sodium alginate^44^ or gelatin.^45^ Wu *et al.* showed that the combination of gelatin and HA provided a suitable moist environment for fibroblasts proliferation and migration.^45^ In another study, Ying *et al.* mixed HA-tyramine with collagen I-hydrobenzoic derivative to obtain a hydrogel with higher glass transition temperature, higher thermal transition temperature and the best wound healing ratio compared to other groups.^19^ However, HA can be chemically modified and conjugated by employing reactive functional groups, such as carboxyl, hydroxyl and acetylamino groups. Among them, functionalization of the primary and secondary hydroxyl groups using alkyl succinic anhydrides or methacrylic anhydride is the common used technique to obtain photocrosslinkable HA.^8,37,38,46^ In previous works, esterification by methacrylic and maleic anhydride, at different degrees of substitution (DS), has been used to synthesize methacrylated (MEHA) and maleated (MAHA) HA materials, respectively.^8,37,47^ In particular, it has been highlighted the possibility to fine control the DS of HA matrix to obtain photocrosslinkable hydrogels with modulated adsorption capability and mechanical properties.^47^ Here, the biocompatibility of MEHA and MAHA hydrogels was explored by *in vitro* and *in vivo* studies to estimate their effect on cell proliferation and tissue inflammation. To achieve this aim, an *in vitro* study was performed by using different cell lines involved in wound healing as human endothelial cells (HUVEC), human dermal fibroblasts (HDF) and human amnion-derived mesenchymal stem cells (HAD-MSC). Furthermore, inflammation study was carried out on murine macrophage cell line to evaluate the influence of both hydrogels on interleukins production. *In vivo* study involved the subcutaneous implantation of both hydrogels in BALB/c mice for up to 28 days and allowed to qualitatively and quantitatively evaluate the inflammatory response at the tissue/implant interface.

## 2. Experimental section

### 2.1. Modified HA synthesis and network preparation

**Fig. 1:**
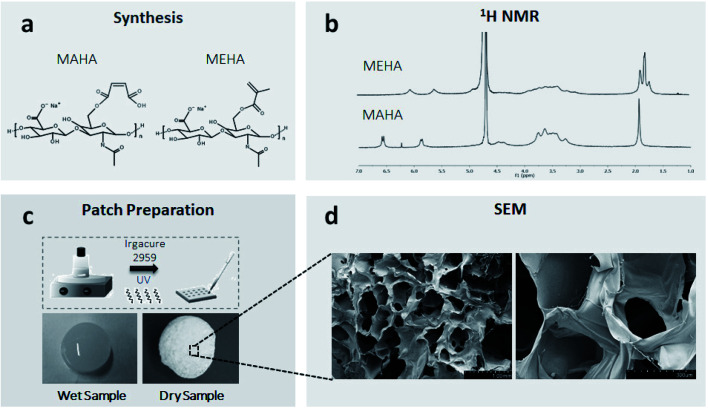
From synthesis to MAHA and MEHA photocrosslinked patches: (a) synthesis of MEHA and MAHA; (b) ^1^H NMR of synthesized materials; (c) photocrosslinking procedure by UV light; (d), SEM images of photocrosslinked and dried samples (scale bars: left – 1 mm, right – 300 μm).

MEHA and MAHA materials were synthesized following a protocol previously described.^8,37,47^ Briefly, hyaluronic acid sodium salt (HAs, *M*_w_ = ∼340 kDa, Bloomage Freda Biopharm Co. Ltd., Shandong, China) was functionalized with methacrylic (ME; Sigma Aldrich) and maleic anhydride (MA, Kelong Chemical Co. Ltd.) to bind photoactive polymerizable moieties on the polymer chain (Fig. 1a). By modulating the synthesis parameters (pH, temperature, molar ratios ME/or MA/HAs) it was possible to obtain high DS for the modified HA polymers.^47^^1^H NMR was used to evaluate the DS of the modified HAs. The solutions of HAs derivatives (both MEHA and MAHA, with different DS) in deuterium oxide (D_2_O) were prepared at a concentration of 5–6 mg mL^−1^ and analyzed at a frequency of 400 MHz using a Bruker AVIII 400HD nuclear magnetic resonance spectrometer (Swiss). To formulate liquid photocrosslinkable resins, MEHA and MAHA were dissolved in distilled water (dH_2_O) solution, at different weight/volume ratio (2 and 3% wt/v respectively), containing 0.1% (wt/v) of 2-hydroxy-4′-(2-hydroxyethoxy)-2-methylpropiophenone (Irgacure 2959, Sigma-Aldrich) as a biocompatible photoinitiator. MEHA and MAHA solutions were moved to a cell culture plate (diameter (*d*) = 10 ± 0.4 cm) and then irradiated with ultraviolet (UV) light (Analytik Jena UVP crosslinker, *λ*: 365 nm, ∼100 μJ s^−1^ cm^−2^) for 60 s and 120 s, respectively (Fig. 1c). Crosslinked samples were punched into round shape discs (*d* = 8.0 ± 0.1 mm) and kept in reverse osmosis water for 24 h. After the extraction, the samples were freeze dried and stored at 4 °C before testing. For the morphological characterization, samples were coated with an ultrathin layer of Au/Pt by using an ion sputter and then observed by scanning electron microscopy (SEM, FEI Quanta FEG 200, the Netherland). Swelling/degradation studies were also performed. To this aim, completely dried hydrogel samples were weighted (*w*_0_) and left to swell in physiological conditions up to 14 days (pH ∼ 7.4, *T* ∼ 37 °C). The swollen hydrogels were then taken out from phosphate-buffered saline (PBS) at fixed time, the surface adsorbed water was removed by filter paper, the weight was recorded (*w*_*t*_) and the samples placed in PBS again. The swelling ratio (*Q*), expressed as mean ± standard deviation, was obtained using the following expression: *Q* = (*w*_*t*_ − *w*_0_)/*w*_0_.

### 2.2. Cell culture

HDF, HAD-MSC and HUVEC (ScienCell – California, USA) were cultured in 75 cm^2^ cell culture flask. HDF were cultured in Dulbecco's modified Eagle's medium (DMEM, Gibco, USA) with 10% fetal bovine serum (FBS), antibiotic solution (streptomycin 100 μg mL^−1^ and penicillin 100 U mL^−1^, Sigma Chem. Co). HUVEC and HAD-MSC were maintained in endothelial cell medium (ECM, 1001, ScienCell) and mesenchymal stem cells medium (MSCM, 7501, ScienCell), respectively. For inflammation study, murine macrophage cell line RAW 264.7 (Sigma-Aldrich, Italy) was used. The cells were used at passage 4–6 and incubated in DMEM supplemented with 10% FBS, antibiotic solution (streptomycin 100 μg mL^−1^ and penicillin 100 U mL^−1^) and 2 mM l-glutamine (Gibco, USA) at 37 °C in a humidified atmosphere with 5% CO_2_ and 95% air. Four disc-shaped hydrogel samples each type were sterilized overnight by soaking in 70% ethanol. After that, samples were rinsed in sterilized phosphate buffered solution (PBS, Sigma-Aldrich) and washed twice, 5 min each and then they were kept with medium for 24 h before seeding the cells.

### 2.3. Cell viability

Cell viability was measured by using Cell Counting Kit-8 (CCK8, MCE-New Jersey, USA). CCK8 quantification was performed under the instruction of product to study the influence of MEHA and MAHA hydrogels on cell viability. Briefly, cells were seeded on materials in 96-well plate at a density of 2000 cells per well in 100 μL medium for 1, 3, 7 days and at each time point, 10 μL of the CCK8 working solution was added and incubated for 4 h in the incubator. Finally, absorbance at 450 nm was measured using a microplate reader (Multiskan™ FC, Thermo, USA).

### 2.4. *In vitro* inflammation study: pro-inflammatory and anti-inflammatory interleukins evaluation

The capability of MEHA and MAHA hydrogels to modulate the inflammation reaction was investigated by measuring the basal levels of pro- and anti-inflammatory interleukins secretion. In particular, the study was performed in physiological conditions on RAW 264.7 at density of 10 000 cells per well. At day 3 of cell culture, interleukin-1β (IL-1β), interleukin-6 (IL-6) and interleukin-10 (IL-10) levels in cell supernatants were quantified using ELISA kits (Affymetrix Italia, Srl) following the manufacturer's instructions. The absorbance was measured at 450 nm by VICTOR Multilabel plate reader.

### 2.5. Animals and *in vivo* subcutaneous implantation

Male BALB/c mice, ranging from six to ten weeks old, obtained from the Experimental Animal Center of Sun Yat-sen University, were used for the experiments. Mice were randomly divided into 3 groups (MEHA, MAHA and control). Thirty mice in each group were anesthetized with isoflurane, the hair in the dorsum was removed and the surgical area was prepared with aseptic technique. The subcutaneous incision was made in the dorsum of each mice and materials were placed laterally 15 mm on each side of the incision. Incisions were closed by sutures and mice were monitored for up to 28 days. For the control group, incisions were closed by sutures without any implantation. Images were taken by Canon camera at day 1, 3, 7, 14, and 28. After 28 days, mice were sacrificed and the surrounding tissues were for histological and immunohistochemical analyses.

### 2.6. Histological and immunohistochemical evaluation

At day 1, 3, 7, 14, and 28 post-implantations, surrounding tissues including implanted materials were excised and stored in formalin-fixing and paraffin-embedding for histological and immunohistochemical evaluation. Tissue was split in two units and three-micrometer-thick sections were sliced using a Leica Cryo-stat (CM1850) and placed on poly-l-lysine coated slides. To evaluate the tissue responses to the materials, cell infiltration of the different tissue layers and angiogenesis was visualized by hematoxylin–eosin (H&E) staining. Furthermore, to determine the extent of biomaterial-induced inflammatory reactions, immunohistochemical (IHC) analyses for CD11b+ inflammatory cells were performed on tissue slices. CD11b used in this study was purchased from Abcam (1 : 1000; ab133357, Abcam Inc., Cambridge, MA, USA). Before IHC staining, antigen retrieval step was performed. Thereafter, slices were washed twice in PBS and then blocked with 1% Bovine Serum Albumin (BSA, Sigma Aldrich) solution by incubating for 40 min at 37 °C. After rinsing in PBS, tissue sections were blocked in 1% goat serum (BOSTER, China) for 1 hour at room temperature and then incubated with the primary antibody CD11b diluted in 1% BSA for 2 h at 37 °C. HRP conjugate was used for detection. The density of inflammatory cells around the implant was assessed using immunohistochemistry. Cell density was calculated as the number of positive cells per area, approximately similar areas were used in each case calculated from a commercial imaging software (ImageJ, National Institutes of Health, Bethesda, MD). Stained sections were visualized using a digital slide scanning system (AxioScan.Z1, Carl Zeiss AG).

### 2.7. Statistical analysis

All data was presented as mean ± standard deviation. One-way or two-way ANOVA followed by Turkey's post-hoc test or two-tailed Student's *t*-test, as appropriate, was used to analyze the statistical significance. *p* < 0.05 was considered as statistically significant.

## 3. Results and discussion

### 3.1. Synthesis of modified HA and network preparation

The DS of HA was obtained from ^1^H NMR spectra reported in Fig. 1b. In particular, it was determined from the ratio of the peak areas corresponding to the methacrylate (5.6 and 6.1 ppm) and maleated (5.9 and 6.6 ppm) moieties with the protons of methyl (–CH_3_ – 1.9 ppm) that belong to the *N*-acetyl group and served as the reference.

By optimizing the reaction conditions, it has been possible to obtain MEHA and MAHA with high DS of 79.96 ± 2.49 and 85.49 ± 4.86, respectively. The functionalized HA with higher DS represented the best compromise in terms of physico-chemical and mechanical properties with an elastic modulus of 20.1 kPa and 41.2 kPa for MAHA and MEHA, respectively.^47^ Moreover, SEM analysis of the dried samples showed an open pore architecture (Fig. 1d) with an average pore size ranging from 300 to 600 μm, which showed to be suitable to influence the cellular fate as it can affect molecular diffusion of oxygen, nutrients and other water-soluble metabolites, cell colonization, and water swelling.

**Fig. 2:**
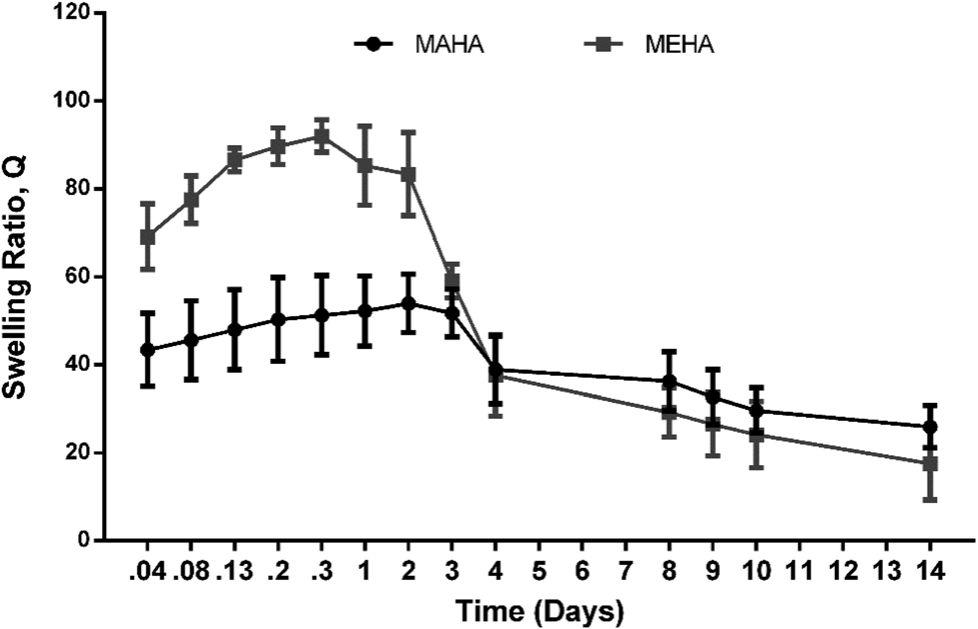
Swelling and degradation behavior of MEHA and MAHA at different time points until 14 days in physiological conditions. “*Q*” is expressed as mean value ± standard deviation (*n* = 3).

Fig. 2 shows the swelling/degradation behavior of MEHA and MAHA in PBS at physiological conditions after 14 days. Both hydrogels were quite stable in the first 72 h, even though MEHA hydrogel showed a slight reduction of the swelling ratio already after 48 h. This behavior could be ascribed to an initial degradation of MEHA material, which clearly appeared at 7 days with a drastic reduction of “*Q*” value. Meanwhile, a different behavior was observed for MAHA where the swelling ratio increased up to 3 days while after 7 days, the material slightly started degrading, highlighting a reduction of the “*Q*” value. However, even though MAHA samples were characterized by lower structural properties, compared to MEHA hydrogels, they tended to maintain their shape over time. In particular, MAHA behavior is similar to hyaluronic acid grafted pullulan polymers (HA-*g*-Pu) that was developed by Li *et al.* as biocompatible wound healing film.^41^

### 3.2. Cell proliferation in MEHA and MAHA

**Fig. 3:**
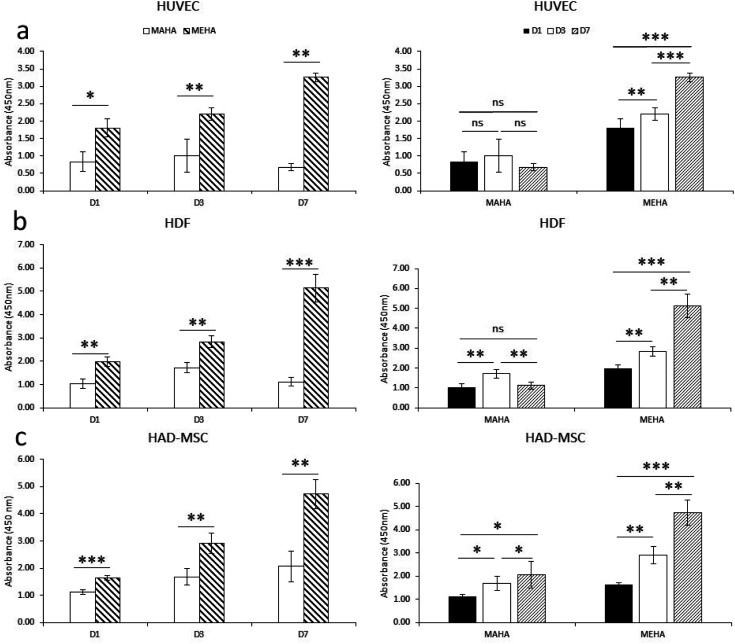
Cell proliferation investigation of HUVEC (a), HDF (b) and HAD-MSC (c) seeded on MEHA and MAHA at long time (1, 3 and 7 days). Cell proliferation was performed by CCK8 assay using manufacturer's protocol. *, *p* < 0.05; **, *p* < 0.01; ***, *p* < 0.0001; ns, no significant difference.

Biomaterials, especially for implanted tissue engineering, must have the ability to promote cell adhesion and maintain the cell biological function. In particular, among natural hydrogels, HA shows great appealing for biomedical applications because represents the natural polysaccharide of ECM with good biocompatibility. Here, modified-HA patches were developed in order to modulate their physico-chemical properties. To assess the effects of these modified-HA (MEHA and MAHA) on cell viability and proliferation, some cells as HUVEC, HDF and HAD-MSC involved in wound healing were analyzed. The results demonstrated a significant higher cell viability and proliferation in MEHA than in MAHA (Fig. 3a–c) with regard to three types of cells over the culture time. In particular, the results highlighted that MAHA did not induce the cell proliferation over the culture time but it had no visible cytotoxic effect. This behavior may be probably ascribed to the lower mechanical properties of the maleated HA if compared with the methacrylated derivative.^8,37,47^

Indeed, as demonstrated by Burdick *et al.*, methacrylated HA hydrogels showed improved rigidity and good degradation profile, compared to other HA hydrogels, while maintaining good biocompatibility.^46^ Furthermore, Poldervaart *et al.* demonstrated that methacrylated-HA hydrogels showed a good primary cell survival (hMSC) for long time until day 21,^29^ and in contrast to other hydrogels, the presence of bioactive peptides (*i.e.* RGD) is not necessary to improve the hydrogel's performance.^48^

### 3.3. Inflammation response on *in vitro* murine macrophage cell culture

**Fig. 4:**
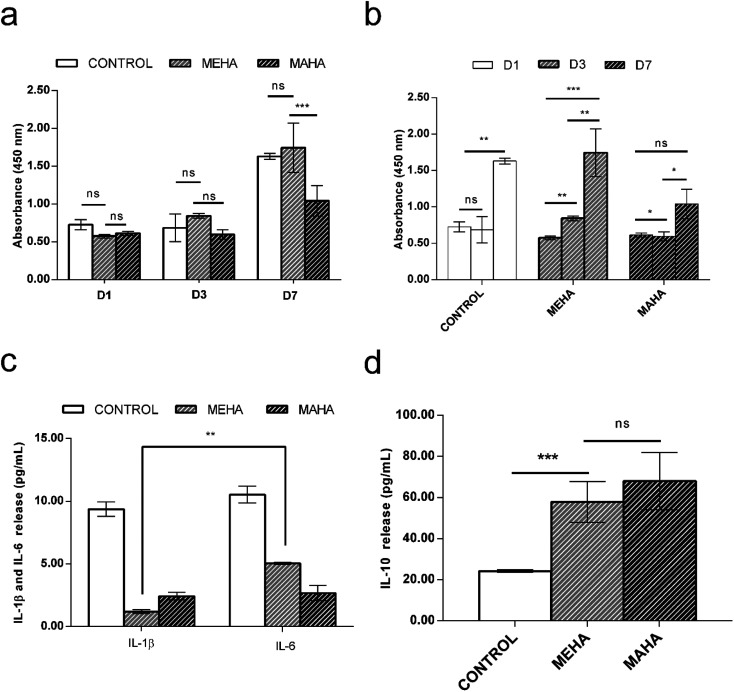
Effect of MEHA and MAHA hydrogels on RAW 264.7 in terms of: (a) and (b) cell proliferation after 1, 3 and 7 days of cell culture. Macrophage proliferation was performed by CCK8 assay using manufacturer's protocol; (c) and (d) interleukin-1β (IL-1β), interleukin-6 (IL-6) and interleukin-10 (IL-10) levels in basal condition. For (c) and (d), measurements were performed after 3 days of cell–material interaction and results (expressed as picograms per mL) are reported as mean ± standard deviation of 4 independent experiments. *, *p* < 0.05; **, *p* < 0.01; ***, *p* < 0.0001; ns, no significant difference.

The use of biomaterial-based medical devices may activate the acute inflammatory pathway by the release of specific mediators such as interleukins. In particular, acute inflammatory response is characterized by the production of pro-inflammatory cytokines such as IL-1β, which are involved in the recruitment of cells by the immune response.^49^ Here, the *in vitro* behavior of modified-HA on inflammatory response was investigated. In particular, MEHA hydrogels induced an increasing of macrophage cell proliferation over culture time with significant difference between 1 and 7 days (*p* < 0.0001), whereas MAHA hydrogel did not support the macrophage proliferation over the culture time (Fig. 4a and b). This behavior was confirmed by *in vitro* interleukins secretion. Indeed, higher secretion levels of the multifunctional pro-inflammatory cytokine IL-6 was observed in MEHA (5.05 ± 0.09 pg mL^−1^) than MAHA hydrogel (2.67 ± 0.60 pg mL^−1^) (Fig. 4c) in basal condition. Furthermore, only for MEHA, it is possible to observe a significant difference (IL-6 > IL-1β) in the production of pro-inflammatory cytokines. Moreover, MEHA and MAHA materials highlighted *in vitro* good anti-inflammatory properties as evidenced by high levels of IL-10 which resulted 57.92 ± 9.87 pg mL^−1^ and 68.08 ± 13.94 pg mL^−1^ without significant difference, respectively (Fig. 4d).

### 3.4. *In vivo* biocompatibility

**Fig. 5:**
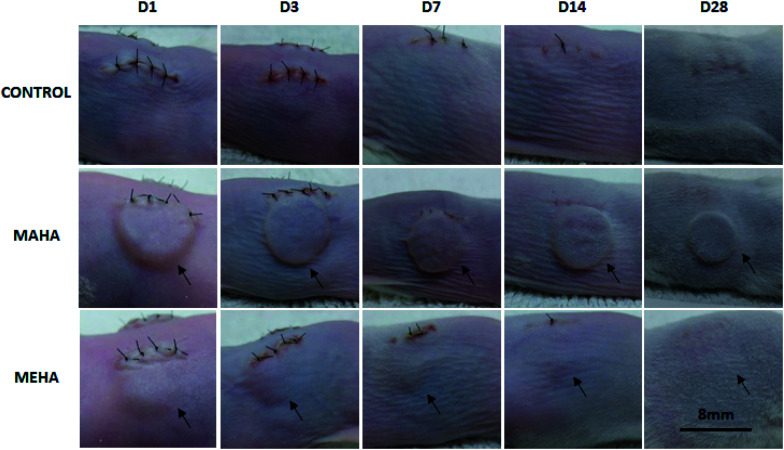
Appearance of the skin in the implantation site. No inflammatory reactions including erythema, swollen, infiltration and blister were observed during the 28 days of implantation. Images were obtained by means of Canon camera at different time points. Black arrows indicate MAHA or MEHA implantation site. Scale bar: 8 mm.

**Fig. 6:**
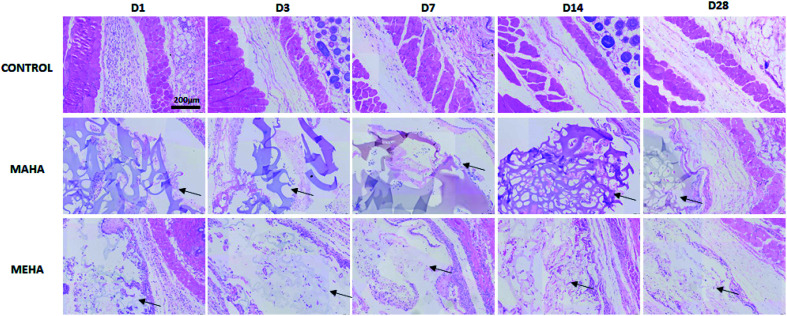
H&E staining of tissues surrounding different implants at day 1, 3, 7, 14, 28 after implantation. MAHA and MEHA were observed in the implantation site, indicated by black arrows. Scale bar: 200 μm.

Given that MAHA and MEHA did not show any significant *in vitro* cytotoxic effects on HDFs, HAD-MSCs, HUVECs and particularly, MEHA could promote cell proliferation, they were subcutaneously implanted in BALB/c mice for up to 28 days to investigate the *in vivo* inflammation response. The appearance of the skin in the implantation site was observed and images were taken on day 1, 3, 7, 14 and 28 (Fig. 5). No skin reactions including erythema, swollen, infiltration, blister and ulcer, were observed in the MAHA and MEHA implanted sites and suture around the incision side was off after day 7, and totally healed after day 14. At day 28, no difference was captured in the skin appearance in the implanted area in all groups. Furthermore, HE staining showed the presence of MAHA and MEHA at 28 days after *in vivo* implantation. The results indicated that both MAHA and MEHA had no severe inflammation response to the implanted site skin (Fig. 6).

### 3.5. Inflammatory cells and angiogenesis to the implantation site

**Fig. 7:**
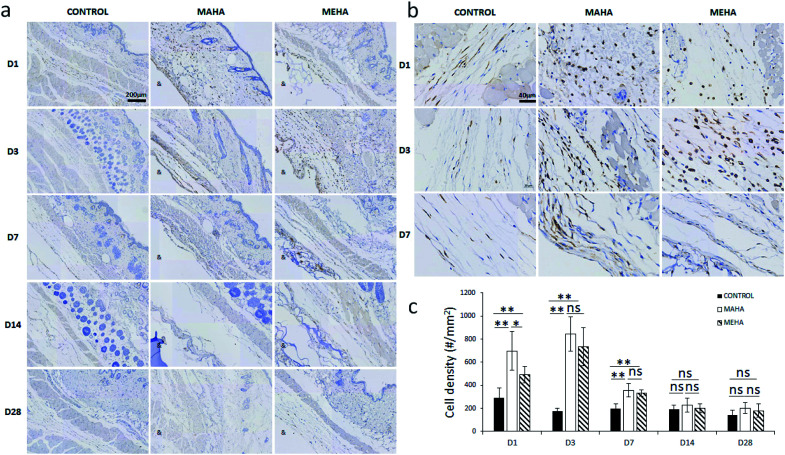
Immunohistochemical staining of tissues surrounding different implants at day 1, 3, 7, 14, 28 after implantation. (a) Qualitative evaluation, scale bar: 200 μm; (b) magnification of the IHC image, scale bar: 40 μm; (c) quantitative assessment of CD11b+ cell density around the implants. “&” indicates the implantation area. “#” indicates cell number. *, *p* < 0.05; **, *p* < 0.01; ns, no significant difference.

The main challenge of tissue engineering is to create a tissue substitute able to address the limitation concerning the diffusion of nutrients and wastes. Indeed, the failure of engineered tissue is due to the cell death and hypoxia. In this way, the stimulation of blood vessel growth is expected to assure tissue survival and the *in vivo* success of the engineered tissue construct. The formation of vascular network depends on angiogenesis that is the result of a biochemical cascade of angiogenic factors. Only few biomaterials show pro-angiogenic activity due to their chemistry, one of that is represented by hyaluronic acid.^50^ This activity may be ascribed to surface chemistry that could stimulate a modulated inflammatory response. In this way, here histological analysis was performed to evaluate the infiltration of inflammatory cells and angiogenesis into the subcutaneous implants at different time points. IHC staining (Fig. 7a–c) was carried out to determine the distribution and the density of inflammatory cells (CD11b+). Results showed more invaded cells around MEHA and MAHA implants than control group at day 7. In particular, an accumulation of inflammatory cells in both MAHA and MEHA implanted tissue was observed (Fig. 7a and b).

**Fig. 8:**
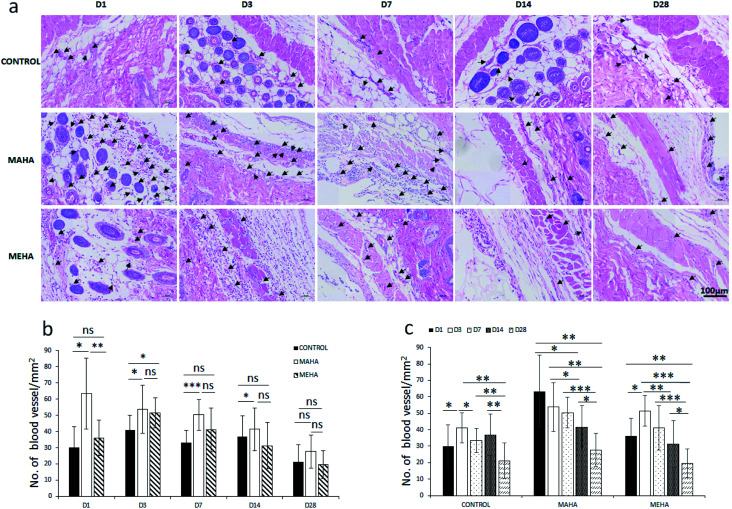
Blood vessel quantification in tissues surrounding different implants at day 1, 3, 7, 14, 28 after implantation. (a) Qualitative evaluation; (b) and (c) quantitative assessment of blood vessels around the implants. *, *p* < 0.05; **, *p* < 0.01; ***, *p* < 0.0001; ns, no significant difference. Black arrows indicate blood vessel. Scale bar: 100 μm.

The density of CD11b+ cells at the tissue/material interface reached a maximum value at day 3 after implantation, while the control group showed a reduced number of inflammatory cells (Fig. 7c, *p* < 0.01). There was a higher number of CD11b+ cells in MAHA group comparing to MEHA at day 1 cells (Fig. 7c, *p* < 0.01), while no significant difference was observed between MAHA and MEHA group at day 3, which mainly caused by the implantation practice. Thereafter, the number of CD11b+ cells decreased at day 7; meanwhile no significant difference was noticed after 14 days of implantation. In particular, MAHA and MEHA induced angiogenesis immediately after implantation (Fig. 8). However, highest angiogenesis was observed at two different time point as day 1 and day 3 for MAHA and MEHA, respectively. During the observation period, the number of blood vessels decreased and no significant difference was evident at day 28 in both MAHA and MEHA groups. In MEHA group, a similar phenomenon was observed once implanted, while the ability to stimulate angiogenesis was not as strong as MAHA. In brief, no significant chronic inflammation reaction was highlighted in both MEHA and MAHA in the long-term implantation, opening new fascinating perspectives of these appealing materials as potential candidates for tissue engineering applications.

## 4. Conclusion

Modified-HA based hydrogels have gained research attention as UV photocrosslinkable materials to be used as matrix to produce substrates (*i.e.* patches) and/or scaffolds with interesting physico-chemical and biological properties. The purpose of this study was to assess the influence of two different chemical modifications (methacrylic and maleic groups) on the cell viability, proliferation and tissue inflammation. In this way, MEHA and MAHA hydrogels were cultured with HUVEC, HDF and HAD-MSC, examining the inflammation chemokines and inflammation response *in vitro* and *in vivo* models. The results showed that MEHA significantly promoted HDFs, HAD-MSCs and HUVECs proliferation compared to MAHA. Additionally, both MAHA and MEHA showed anti-inflammation ability on *in vitro* cell culture. The *in vivo* study indicated that they have no significant inflammation stimulation in long-term implantation, while they induced angiogenesis in the 3 days. The overall results suggested that modified-HA based hydrogels are biocompatible, versatile and promising materials for biomedical application and their specific biological properties could be used to develop different advanced biomedical products for tissue engineering. Furthermore, the results highlighted the potential of these materials as wound dressings as they were able to provide a moist environment and protection from infections. However, future studies will be devoted to the analysis of their capability to act as skin barrier, reduce skin wound infections, eliminate wound exudates and stimulate skin regeneration by wound closure with an aesthetically acceptable scar. Therefore, using an appropriate antibacterial method could be also another interesting strategy to accelerate wound healing.

## Ethical statement

All animal procedures were performed in accordance with the Guidelines for Care and Use of Laboratory Animals of the First Affiliated Hospital, Sun Yat-sen University and approved by the Animal Ethics Committee of the First Affiliated Hospital, Sun Yat-sen University. Furthermore, significant efforts were made to minimize both the number of animals used and their respective suffering.

## Conflicts of interest

Authors declare no conflict of interest related to this study.

## Supplementary Material
